# Vav2 protein overexpression marks and may predict the aggressive subtype of ductal carcinoma in situ

**DOI:** 10.1186/2050-7771-2-22

**Published:** 2014-11-28

**Authors:** YunQing Jiang, Indira Prabakaran, Fei Wan, Nandita Mitra, Dana K Furstenau, Rupert K Hung, Siyuan Cao, Paul J Zhang, Douglas L Fraker, Marina A Guvakova

**Affiliations:** Division of Endocrine and Oncologic Surgery, Department of Surgery, Perelman School of Medicine, University of Pennsylvania, Philadelphia, PA USA; Department of Biostatistics and Epidemiology, Perelman School of Medicine, University of Pennsylvania, Philadelphia, PA USA; Department of Pathology and Laboratory Medicine, Perelman School of Medicine, University of Pennsylvania, Philadelphia, PA USA

**Keywords:** Ductal carcinoma *in situ*, Breast, Vav2, Rap1, Insulin-like Growth Factor I receptor

## Abstract

**Background:**

A subset of patients with ductal carcinoma *in situ* (DCIS) will develop invasive breast cancer (IBC). To date, there are no effective predictive biomarkers for identifying this subset with worse prognosis whose lesions are essentially indistinguishable histologically from those with favorable outcomes. We hypothesized that measurable parameters that discriminate DCIS from DCIS with concurrent invasion may serve as diagnostic biomarkers (BM) of progressive cancer *in situ* (CIS).

**Results:**

Using a novel imaging-based method of tissue testing, we measured the relative expression levels of three candidate BM proteins specifically implicated in IBC progression - the insulin-like growth factor I receptor (IGF-IR), Ras-related protein 1 (Rap1), and Vav2 oncoprotein. Protein profiles were compared in 42 histologically normal mammary epithelial samples, 71 CIS (35 without/36 with invasion either on diagnostic biopsy or final surgical excision), and 98 IBC of known estrogen receptor (ER), progesterone receptor (PR) and human epidermal growth factor receptor 2 (HER2) status. The levels of the IGF-IR and Rap1 protein expression were significantly elevated in ER-positive (ER+/PR+/-/HER2 –) DCIS relative to normal epithelium (*P* <0.0001). The IGF-IR protein expression was also significantly up regulated in HER2-positive (ER+/-/PR+/-/HER2+) DCIS relative to normal epithelium (*P* = 0.0002). IGF-IR and Rap1 protein expression levels were similar among DCIS patients without or with concurrent invasion. Vav2 upregulation in DCIS relative to normal group was not associated with steroid hormone receptor and HER2 status, but was associated with the presence of concurrent invasion, including microinvasion (invasive foci of less than 1 mm). DCIS with high Vav2 were more than twice as likely to progress to invasive cancers as DCIS with low Vav2 (odds ratio, 2.42; 95% CI, 1.26-4-65; *P* =0.008). Furthermore, a receiver operating characteristic curve analysis revealed moderate ability of Vav2 protein expression measurements in DCIS to predict the existence of invasion concurrent with DCIS (area under the curve, 0.71; 95% CI, 0.59- 0.84).

**Conclusions:**

Our novel findings hold promise for utilizing Vav2 protein as a predictive BM for differentiating progressive from non-progressive DCIS.

**Electronic supplementary material:**

The online version of this article (doi:10.1186/2050-7771-2-22) contains supplementary material, which is available to authorized users.

## Background

Ductal carcinoma *in situ* (DCIS) of the breast is a noninvasive lesion most commonly detected in asymptomatic women as a small area of abnormal calcification on mammography. Incidence of DCIS has risen rapidly over the past decades largely due to increased mammography screening [1]. Currently, DCIS accounts for nearly one-fourth of all new breast cancer (BC) diagnoses, with more than 1 million women in the United States projected to be diagnosed with DCIS by 2020. Routine screening cannot reliably distinguish progressive and indolent DCIS. Hence, although surgery is still considered the standard treatment for patients diagnosed with DCIS, there has been considerable debate in clinical practice regarding the classification of these lesions [2].

To date, little is known about molecular biomarkers (BM) that may help to determine the likelihood that DCIS identified on diagnostic biopsy would remain contained *in situ* or become invasive [3]. The hallmark of progressive tumors is the abnormal migratory properties of tumor cells and their abilities to extend beyond the original tumor site. Our candidate BMs, the insulin-like growth factor I receptor (IGF-IR), Ras-related protein 1 (Rap1), and oncoprotein Vav2, are molecules whose up-regulation have been implicated in promoting the aggressive behavior of cancer cells in preclinical models [4–6].

As the key receptor in cancer cell proliferation and migration, IGF-IR has become one of the most intensively investigated molecular targets in oncology [4–6]. In our previous studies, the concurrent up-regulation of the IGF-IR and the small GTPase Rap1 in primary BC suggested the involvement of both proteins in the etiology of the disease [7]. Rap1 is highly homologous to the small GTPase Ras, whose oncogenic form plays a critical role in promoting cancers [8, 9]. Although oncogenic mutations of Rap1 have not been found, Rap1 deregulation in cancer may occur following abnormal regulation of the hormone, growth factor, and/or cytokine receptors [10–12]. In the human BC model, hyperactivation of Rap1 was related to loss of mammary epithelial cell polarity, cell invasion *in vitro* and tumorigenicity in nude mice [13]. Our biochemical studies showed that downstream inputs from the activated IGF-IR to Rap1 promoted BC cell migration [14]. Hence, we hypothesized that up-regulation of IGF-IR/Rap1 may increase the propensity of DCIS transitioning to IBC.

The overexpression of oncoprotein Vav2 has been implicated in advanced metastatic breast cancer [15]. As a member of the Vav guanine nucleotide exchange factor family of oncogenes, Vav2 activates the small Rho family GTPases (RhoA, Rac, Cdc42) that may promote cell migration by altering cell morphology and gene expression [16]. The Vav2 protein acts downstream of a myriad of cell surface receptors, many of which are overexpressed already in precancers and therefore may activate Vav2 to drive cancer progression [17]. Despite literature implicating a potential role of Vav2 protein in IBC progression, studies on human tissue supporting this hypothesis are largely missing.

In this study, we applied our imaging-based analytic tools to accurately quantify, on continuous-scale levels, three candidate BM proteins in biopsy and surgical specimens from 144 patients diagnosed with BC. We based our study on 42 histologically normal mammary epithelial samples from patients with BC, 71 CIS (35 without/36 with invasion either on diagnostic core needle biopsy (CNB) or final surgical excision), and 98 IBC with the aim of identifying associations between protein expression in DCIS and the presence of invasion concurrent with DCIS. We provide updated results for IGF-IR and Rap1, making the conclusions considerably more definitive. In addition, we describe novel results for Vav2 protein, whose protein expression in early stage breast cancer had not been investigated.

## Materials and methods

### Patient samples

211 breast specimens, collected from patients who had surgical excision for BC between 2007 and 2013, include 42 histologically normal tissues; 71 CIS (35 without invasion; 11 associated with microinvasion <0.1 cm; 25 associated with invasion >0.1 cm), and 98 IBC (76 invasive ductal carcinoma (IDC) and 22 invasive lobular carcinoma (ILC) as summarized in Table 1. CIS samples, DCIS and lobular carcinoma *in situ* (LCIS), were identified either on diagnostic CNB (65%) or surgical excisions (35%). Three patients with DCIS diagnosed on CNB had chemotherapy prior to surgery; for patients with de-identified tissue samples information on systemic therapy was unavailable. The presence of DCIS was based on primary diagnosis and verification by the pathologist (P.J.Z.) prior to cutting slides for analysis. The characteristics of the analyzed formalin-fixed paraffin-embedded (FFPE) tumors are presented in accordance with the REMARK recommendations [18]. Standard prognostic variables, tumor size, lymph node, grade, as well as human epidermal growth factor receptor 2 (HER2), estrogen receptor α (ER) and progesterone receptor (PR) status from pathology reports are summarized in Table 2. Note, the erbB-2 immunocytochemical assay was performed on DCIS and IBC using DakoCytomation (HercepTest) kit. In this study, tumors positive for ER (ER+), positive or negative for PR (PR+/-), and negative for HER2 (HER2-) were classified as ER-positive. Tumors positive for HER2/ErbB2/neu (3+ in >30% positive cells and/or normal gene copy number by fluorescence *in situ* hybridization) were classified as HER2-positive. A score of 2+ in any portion of the tumor cells and 3+ in less than 30% of tumor cells were considered negative/equivocal results. Tumors negative for ER (cutoff <10% positive tumor cells), PR (cutoff <10% positive tumor cells), and HER2 (0, 1+, or 2+ on immunohistochemistry and/or normal gene copy number by fluorescence *in situ* hybridization) were classified as triple negative (TN) breast cancer. A waiver of written documentation of consent was granted; the University of Pennsylvania Institutional Review Board committee had approved the analysis of patients’ tissues and records. The Department of Pathology and Laboratory Medicine at the University of Pennsylvania provided CNB containing DCIS. Residual de-identified human breast tissue was obtained from the University of Pennsylvania Tumor Tissue and Biospecimen Bank (TTAB) and the Cooperative Human Tissue Network (CHTN, Philadelphia, PA).

**Table 1:** **Characteristics of the study population and tissue groups**

Patient tissue groups	No. of samples (%)	Patient age (years)	
		Mean (Median)	Range
Normal^a^	42 (100%)	55.6 (53)	29-85
CIS without invasion	35 (100%)	56.0 (58)	35-80
DCIS	34 (97.1%)	56.1 (58)	35-80
LCIS	1 (2.9%)	52.0 (52)	52-52
CIS with invasion	36 (100%)	54.1 (55)	29-74
DCIS/T1mic	11 (30.6%)	54.5 (55)	38-74
DCIS/IDC	21 (58.3%)	53.3 (55)	29-72
LCIS/ILC	4(11.1%)	57.0 (59)	47-63
IBC	98 (100%)	57.8 (57)	28-88
IDC	76 (77.5%)	56.4 (56)	28-88
ILC	22 (22.5%)	62.6 (62)	32-87

^a^Histologically normal breast epithelium from patients with breast cancer.

**Table 2:** **Clinical characteristics of the study tumor samples**

Characteristic	Status	IBC (%)	CIS (%)
Tumor size	pT1	13.3	
	pT2	65.3	
	pT3	19.4	
	pT4	1.0	
	No report	1.0	
Lymph node status	pN0	44.9	
	pN1	20.4	
	pN2	19.4	
	pN3	8.2	
	pNx	6.1	
	No report	1.0	
Grade (Nottingham score)	I	2.0	4.2
	II	38.8	43.7
	III	46.9	38.0
	No report	12.2	14.1
Estrogen Receptor	Negative	33.7	32.4
	Positive	65.3	67.6
	No report	1.0	0.0
Progesterone Receptor	Negative	43.9	39.4
	Positive	55.1	60.6
	No report	1.0	0
HER2/ErbB2/neu	Negative/ Equivocal	81.6	69.0
	Positive	17.3	21.1
	No report	1.0	9.9

### Immunohistochemistry (IHC)

Methods for IGF-IR and Rap1 IHC staining had been optimized [7]. To detect specific proteins, slides were incubated with commercially tested rabbit polyclonal antibody (Ab) recognizing human IGF-IR β (C-20), Rap1 (121) and Vav2 (H-200) purchased from Santa Cruz Biotechnology. The anti- Vav2 Ab was validated in positive/negative human tissue controls (Additional file 1: Figure S1).

### Quantitative in tissue protein profiling

To minimize subjectivity of the visual assessment of the intensity of IHC staining and compare the relative protein expression levels in IHC stained tissues, we used our imaging-based uniplex (IBU) method previously developed and validated [7]. The measurements were performed in the areas of interest following digital tissue segmentation. The output variable is relative pixel intensity, a ratio calculated by dividing the mean pixel intensity in tissue area containing stroma (value ranging from 0 to 65,535) by the mean pixel intensity of equal area containing cells of interest (value ranging from 0 to 65,535). This computerized method reduces observer-related bias as calculations of relative (rather than absolute) intensities of protein staining minimizes day –to –day fluctuations in IHC results. Multiple repeated measurements (5 < n < 26) of relative intensity of IHC staining in each tissue sample were analyzed.

### Statistical analysis

To compare the differences in protein expression of IGF-IR, Rap1, and Vav2 among different groups of tissues including normal, CIS, IBC and combinations of these groupings, we averaged multiple measurements from each patient and used one-way ANOVA. Post-hoc t-tests were performed for pair-wise comparisons among tissue type groupings and multiple testing corrections were applied using Tukey’s significance test. We further fitted a repeated measures mixed effects model over un-aggregated data with multiple replicates for each patient. A compound symmetric covariance structure was specified to account for the correlation among replicates from each patient. To explore the correlation between various continuous prognostic variables and average protein expression of IGF-IR and Rap1, we computed Pearson’s correlation coefficients. We also fitted univariate logistic regression models using protein expression of IGF-IR, Rap1, and Vav2 as the continuous predictors and binary prognostic variables (HER2, ER, PR) as the dependent variables. Odds ratios (OR) per standard deviation increase and 95% confidence intervals (95% CI) were computed. A two-sided significance level of alpha = 0.05 was used for all tests of significance. Receiver operating characteristic (ROC) curves were used to evaluate how well each marker predicted DCIS lesion type. Areas under the curve (AUC) were computed. The modeling process was verified by cross-validation and the C-Index was computed as a marker of overall model predictability; together with 95% bootstrap CI. All analyses were carried out in SAS version 9.1 (SAS Institute Inc., Cary, NC, USA).

## Results

### Vav2 protein levels increase significantly during the transition from CIS to IBC

**Figure 1:**
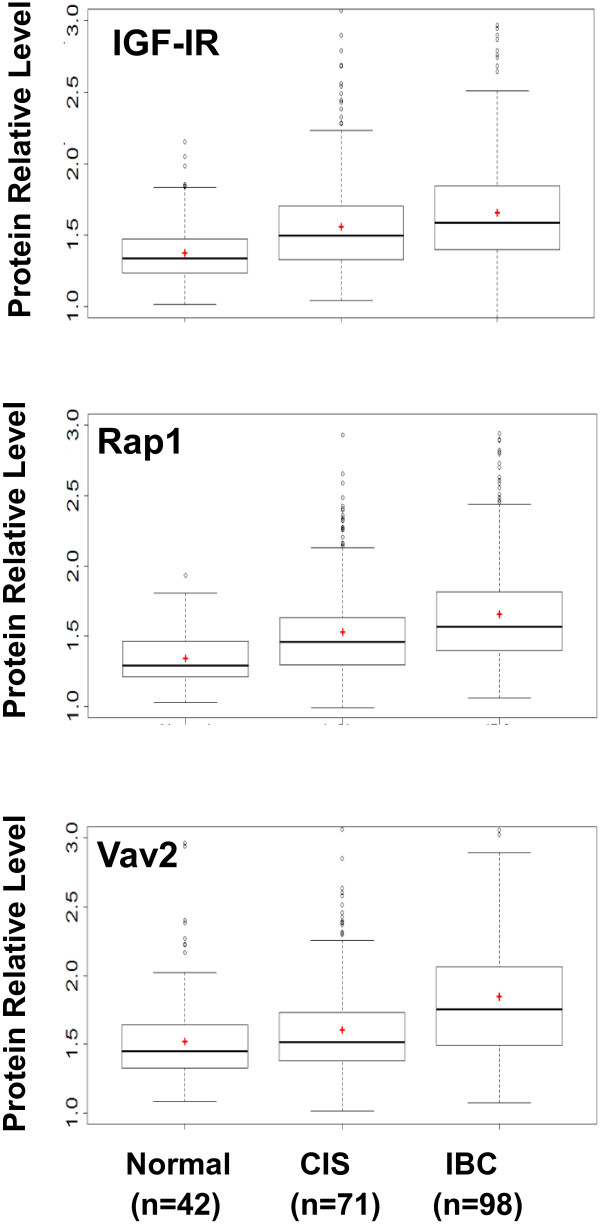
**Box plots for unaggregated measurements of IGF-IR, Rap1, and Vav2 in three major histological groups of tissue: normal mammary epithelium, CIS and IBC.** n, number of samples per group. +, mean value. Horizontal line, median. Boxes, 25^th^ and 75^th^ percentile. Whiskers, 75^th^ +1.5 x IQR and 25^th^ -1.5 x IQR. Dots, group outliers. Y axis, protein levels in relative units.

We first compared protein expression profiles in three histologically different groups of tissue: normal, CIS and IBC, collected from age-matched groups of patients. As shown in Figure 1, the levels of expression of all three proteins were significantly higher in the IBC group compared with the normal group (*P* <0.0001). Higher levels of IGF-IR were expressed in the CIS group than in the normal group (*P* <0.0001), though levels of IGF-IR protein expression were similar between the IBC and CIS groups (*P* = 0.06). Rap1 protein expression was significantly up-regulated in the CIS group compared to the normal group (*P* <0.0001), and in the IBC group compared to the CIS group (*P* = 0.04). In marked contrast, the levels of the Vav2 protein in the CIS group were similar to that in the normal group (*P* = 0.11), whereas significantly higher levels of Vav2 protein expression were seen in the IBC group compared to the CIS group (*P* <0.0001). Thus, significant up-regulation in IGF-IR and Rap1 expression may occur as early as in noninvasive CIS, whereas up-regulation of Vav2 is likely to occur later on during the transition from CIS to IBC.

### High expression of Vav2 in IBC is not associated with steroid hormone receptors (HR) and HER2 status

We next collected clinical characteristics of our study tumor samples to address the question of whether increased IGF-IR, Rap1, and Vav2 protein expression in IBC correlates with standard clinical prognostic factors (patient’s age, tumor size, grade, and lymph node status, HR and HER2 status) and to compare with data from our previously published report [7]. In this study, we have more than doubled the number of analyzed primary IBC samples (n = 98 vs. n = 40) and strengthened our previously reported positive correlation between Rap1 protein expression and tumor’s size (Pearson’s ρ = 0.209; pT_1_-pT_4_; *P* =0.04)*.* No correlations were found between IGF-IR and Vav2 protein levels with the size of the breast tumors. A statistically significant positive correlation was found between IGF-IR protein expression and the patient’s age (Pearson’s ρ = 0.242; 28–88 y; *P* = 0.04) in IDC, but not in ILC (Pearson’s ρ = -0.007; 32–87 y; *P* =0.97). Neither Vav2 nor Rap1 protein expression demonstrated correlation with age at diagnosis of IBC. We did not find statistically significant correlations between IGF-IR, Rap1, and Vav2 protein levels with nodal status or tumor grade. In our cohort of 98 patients with IBC, we confirmed our earlier findings that higher expression of Rap1 showed a statistically significant positive association with both ER and PR positivity (Table 3). IGF-IR was significantly associated with PR positivity (*P* = 0.003), and likely with ER positivity though significance was not achieved for the latter (*P* = 0.11). In marked contrast, higher levels of Vav2 protein expression showed no association with ER + and PR + status in the same group of IBC. There was no association between HER2 positivity and higher IGF-IR, Rap1, and Vav2 protein levels. Thus, in contrast to IGF-IR and Rap1, Vav2 protein expression in IBC does not appear to be associated with ER and PR status.

**Table 3:** **Candidate BM association with steroid HR and HER2 status in IBC**

Candidate BM	Clinical marker	OR	95%	***P*** -value
IGF-IR	ER	1.44	0.92-2.26	0.11
	PR	2.09	1.29-3.38	**0.003** ^**a**^
	HER2	0.94	0.56-1.56	0.81
Rap1	ER	1.85	1.09-3.17	**0.024**
	PR	1.86	1.14-3.05	**0.014**
	HER2	1.17	0.73-1.89	0.81
Vav2	ER	1.09	0.71-1.67	0.69
	PR	1.17	0.78-1.76	0.46
	HER2	0.96	0.58-1.60	0.88

^a^associations between markers found to be significant are in bold.

### Subgroups of DCIS stratified by HR and HER2 statuses have similar levels of Vav2 protein as histologically normal tissue

**Figure 2:**
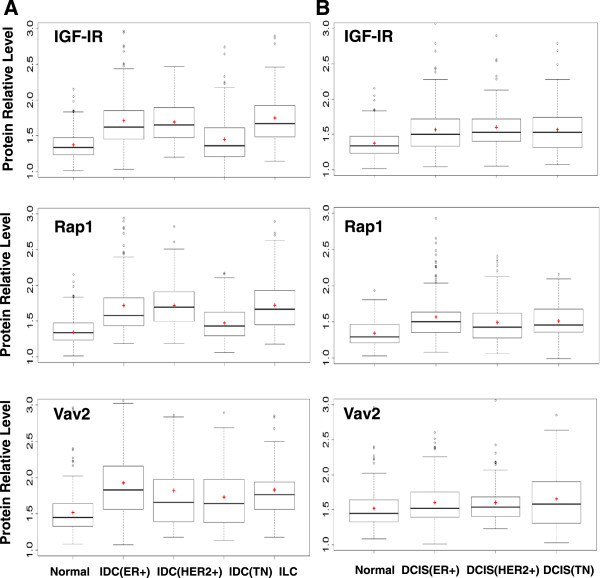
**Box plots for unaggregated measurements of IGF-IR, Rap1, and Vav2 in the tissue groups (normal, ILC) and subgroups of IDC and DCIS. (A)** normal mammary epithelium; ER –positive (ER+), HER2 –positive (HER2+), triple negative (TN) IDC, and ILC; **(B)** normal mammary epithelium; ER –positive (ER+), HER2 –positive (HER2+), triple negative (TN) DCIS. +, mean value. Horizontal line, median. Boxes, 25^th^ and 75^th^ percentile. Whiskers, 75^th^ +1.5 x IQR and 25^th^ -1.5 x IQR. Dots, group outliers. Y axis, protein levels in relative units.

Because IBC and CIS include heterogeneous groups of lesions with diverse morphological and biological features, we posited that the changes in each protein’s expression might evolve differently in different tumor subtypes. We divided the samples of the IDC and DCIS groups into more homogeneous subgroups of tumors based on the available status of HR and HER2 (Table 4). We included ER+/PR+/-/HER2 – tumors in the ER -positive subgroups, ER+/-/PR+/-/HER2+ tumors in the HER2–positive subgroups and ER-/PR-/HER2- tumors in the TN subgroups. We omitted ILC and LCIS samples from further stratification due to small sample size and/or lack of the information required for subgrouping. As illustrated in distributional box plots in Figure 2 and summarized in Table 5, the levels of the IGF-IR and Rap1 proteins were significantly increased in the ER- and HER2 –positive subgroups, but not in the TN subgroup of IDC relative to normal epithelium. IGF-IR and Rap1 protein levels were also significantly up regulated in ILC, 95% of which were ER-positive tumors. Remarkably, a statistically significantly higher expression of Vav2 protein was detected in all tested IDC and ILC compared with normal tissue, regardless of the status of ER and HER2 (Figure 2A). In the DCIS subgroups, IGF-IR protein levels were significantly increased in all except in the TN subgroup, whereas Rap1 protein levels were significantly increased only in the ER-positive subgroup (Figure 2B). Most interestingly, pair-wise comparisons between normal tissue and DCIS subgroups showed that there were no statistically significant differences in Vav2 protein expression among normal tissue and the subgroups of DCIS stratified based on ER and HER2 status (Table 5).

**Table 4:** **Characteristics of patients and tissue subgroups stratified by HR and HER2 status**

Tissue subgroups	No. of samples (%)	Patient age (years)	
		Mean (Median)	Range
DCIS	64 (100%)	55.9 (57)	29-80
DCIS (ER+)^a^	38 (59.4%)	53.9 (54)	29-80
DCIS (HER2+)^b^	15 (23.4%)	55.9 (57)	35-69
DCIS (TN)^c^	11 (17.2%)	62.5 (64)	55-74
IDC	76 (100%)	56.1 (56)	28-88
IDC (ER+)^a^	38 (50.0%)	57.8 (56)	29-88
IDC (HER2+)^b^	14 (18.4%)	53.4 (53)	32-88
IDC (TN )^c^	24 (31.6%)	54.9 (58)	28-77

^a^ER -positive subgroups include ER+/PR+/-/HER2 – tumors; ^b^HER2–positive subgroups include ER+/-/PR+/-/HER2+ tumors; ^c^ TN subgroups include ER-/PR-/HER2-.

**Table 5:** **Pair-wise comparison between normal group and subgroups of IDC, group of ILC and subgroups of DCIS**

Groups	IGF-IR	Rap1	Vav2
	Adjusted	Adjusted	Adjusted
	***P*** -value	***P*** -value	***P*** -value
Normal vs. IDC (ER+)	**<.0001**	**<.0001**	**<.0001**
Normal vs. IDC (HER2+)	**<.0001**	**<.0001**	**0.0146**
Normal vs. IDC (TN)	0.8900	0.0903	**0.0172** ^**a**^
Normal vs. ILC	**<.0001**	**<.0001**	**0.0146**
Normal vs. DCIS (ER+)	**<.0001**	**<.0001**	0.1132
Normal vs. DCIS (HER2+)	**0.0002**	0.0814	0.5853
Normal vs. DCIS (TN)	0.0835	0.0708	0.7756

^a^difference between the groups found to be significant are in bold.

### Vav2 protein levels increase in DCIS with concurrent invasion, but not in DCIS without associated invasion

**Figure 3:**
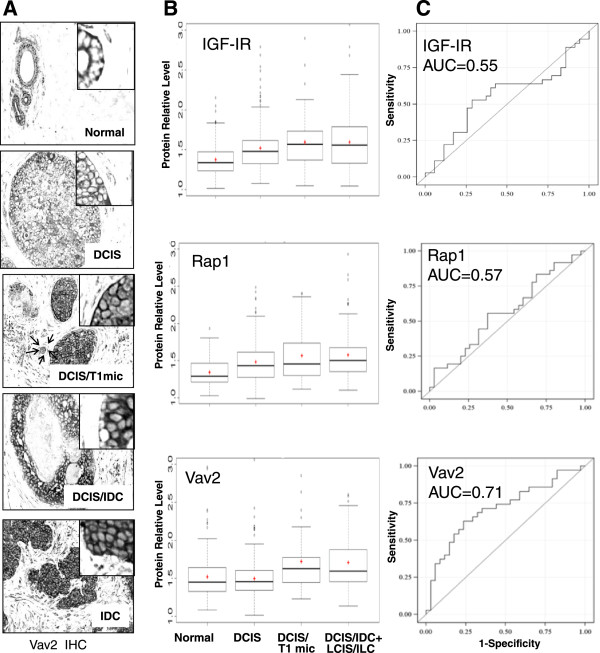
**Differential expression of IGF-IR, Rap1, and Vav2 in DCIS, DCIS with microinvasion, DCIS/LCIS with invasion >1 mm. (A)** Representative monochrome images of Vav2 staining in normal mammary epithelium, DCIS, DCIS/T1mic, DCIS/IDC, and IDC. Original magnification (x200). Insets, enlarged images of epithelial cells. Arrows, suspected microinvasion. **(B)** Box plots for unaggregated measurements of IGF-IR, Rap1, and Vav2 in the groups of tissue: normal mammary epithelium, DCIS, DCIS/T1mic, and DCIS/IDC + LCIS/ILC. +, mean value. Horizontal lines, medians. Boxes, 25^th^ and 75^th^ percentile. Whiskers, 75^th^ +1.5 x IQR and 25^th^ -1.5 x IQR. Dots, group outliers. Y axis, protein levels in relative units. **(C)** The ROC curves for averaged measurements of IGF-IR, Rap1, and Vav2 protein expression.

Because Vav2 was increased in IBC, but not in DCIS lesions, we hypothesized that change in Vav2 protein expression might be associated specifically with the onset of invasive potential in tumor cells, i.e. invasive tumor progression. If so, Vav2 protein up-regulation could be a predictor for the development of invasive potential in tumors without morphologic signs of invasion. To test this hypothesis, we stratified 71 samples of CIS into three histologically different groups with regard to invasion: 35 DCIS without concurrent invasion (DCIS), 11 DCIS associated with microinvasive (<0.1 cm) carcinoma (DCIS/T1mic) and 21DCIS/4LCIS associated with >0.1 cm areas of invasion (DCIS/IDC + LCIS/ILC). It is worth emphasizing that our cohort of DCIS samples are patient- matched cases: DCIS on CNB and subsequent excisions with DCIS without or with microinvasion. This cohort was selected on review of a total of 928 records of DCIS on CNB, with 19.7% of matched cases found to be with microinvasion on subsequent excision, but not on preceding biopsy. While T1mic was not necessarily present on slides that were cut freshly from diagnostic blocks for our analysis, T1mic was documented on CNB or subsequent excision pathology reports. This stringent criterion of selection of DCIS vs. DCIS/T1 mic was applied to stratify DCIS samples, as likely as feasible, into indolent (without invasion) and progressing (with microinvasion) lesions. More importantly, among those cases we only selected DCIS in CNB that involved at least 4 ducts in size to ensure adequate DCIS sampling to represent the disease process and avoid risk of exhausting DCIS tissue for future patient care use. As illustrated in Figure 3A, in normal tissues and DCIS, Vav2 expression was associated with the cell membrane. Vav2 protein was detected on the cell membrane and the cytoplasm in DCIS with invasion and in IDC itself. Remarkably, Vav2 levels in the DCIS group were similar to the normal group (*P* = 0.99), but were increased in DCIS /T1mic and further significantly increased in DCIS/IDC + LCIS/ILC (*P* = 0.03). Rather unexpectedly, compared to normal epithelium, significant increases in IGF-IR (*P* = 0.0025) and Rap1 (*P* = 0.007) were found in earliest proliferative lesions of DCIS without further changes in DCIS/T1mic and DCIS/IDC + LCIS/ILC (Figure 3B). The area under the ROC curve indicates low abilities of IGF-IR and Rap1 measurements to discriminate CIS subgroups (Figure 3C). In marked contrast, Vav2 measurements distinguished CIS with invasion from pure DCIS and normal cells (AUC, 0.71; 95% CI 0.59- 0.84). Moreover, patients that had high levels of Vav2 protein expression in DCIS were more than twice as likely to have concurrent invasion than those with low levels of Vav2 (OR, 2.42; 95% CI 1.26-4-65; *P* = 0.008).

## Discussion

Currently, the most important DCIS factors for clinical decision-making (size and grade) are not necessarily dictated by the biology of DCIS. Comprehensive molecular analyses of DCIS are being limited by the microscopic size of DCIS lesions, the low availability of DCIS samples for laboratory research, and the scarcity of quantitative tools for FFPE tissue examination. In the present study, we identified DCIS on diagnostic CNB and surgical excisions, with stratification by steroid HR status, HER2 status, and the presence of concurrent invasion. We then applied our imaging-based method for direct in-tissue protein quantification, which allowed us to firstly validate our previous findings regarding IGF-IR and Rap1, and secondarily characterize the Vav2 protein expression profiles in patients with breast tumors.

### IGF-IR quantification may aid in determining DCIS sensitivity to hormone and radiation therapy

In this study, we found that significant up-regulation of the IGF-IR protein expression occurred much earlier than previously thought, as early as in DCIS [19, 20]. For patients diagnosed with DCIS and undergoing breast-conserving surgery (BCS), a key decision often is whether to add tamoxifen and /or radiation therapy (RT) after surgery for reducing the risk of local recurrence.

Studies have shown that IGF-IR signalling is a mechanism of escape from hormone dependence that might promote tamoxifen resistance in ER – positive BC [21, 22]. We determined positive associations between the IGF-IR and ER/PR in 98 IBC cases, consistently with early findings [19, 23]. We also determined a statistically significant up-regulation of IGF-IR in the ER-positive subgroup of DCIS, which constitutes 75% of DCIS in general [24] and 59% of DCIS in our study. These novel findings imply that the assessment of IGF-IR levels along with standardized cytomorphological criteria may help to predict tumor response to tamoxifen and perhaps explain why 8% of women taking tamoxifen post-BCS, experience DCIS recurrence or further progression to IBC five years later [25]. IGF-IR is a key receptor in DNA repair and protection against apoptosis. Depletion or inhibition of the IGF-IR has been shown to delay repair of radiation-induced DNA double-strand breaks, enhance tumor radiation sensitivity and amplify RT-induced apoptosis [26–28]. IGF-IR overexpression has been related to resistance to radiation in cell lines and in the clinical setting, making IGF-IR expression a suitable predictive factor for RT response and outcomes [29]. IGF-IR overexpression and activation is also associated with an increased propensity for invasion and metastasis [30]. These IGF-IR effects are mediated by multiple signaling intermediates that influence invasive potential [31]. In this study, we found that both IGF-IR and Rap1 were up-regulated in ER-positive DCIS and IBC. These results suggest that IGF-IR-Rap1 signaling may have a controlling role through the development of most common ER-positive breast malignancy.

Considering experimental evidence of IGF-IR-associated resistance to tamoxifen and radiation, our findings warrant further clinical studies on IGF-IR as potential predictive BM of sensitivity to the most common treatments offered to DCIS patients.

### Vav2 levels may aid in distinguishing indolent from progressive DCIS lesions

In contrast to IGF-IR and Rap1, Vav2 protein expression remained non-elevated in noninvasive proliferative lesions found in the mammary gland such as DCIS until later stages of tumor progression to IBC. To the best of our knowledge, the present report is the first comprehensive study characterizing the clinical relevance of the Vav2 protein for breast pathogenesis.

Earlier IHC attempts reported no difference in Vav2 staining between BC and normal/hyperplastic mammary tissue [32], despite ample preclinical studies implicating oncogenic activation of Vav2 with cancer progression [33–35]. In this study, we found that DCIS in patients whose tumor cells invaded into surrounding tissue had significantly increased levels of Vav2 protein expression compared to those that did not. Of particular interest, the elevated levels of the Vav2 protein were detected in DCIS/T1 mic, although concurrent microinvasion was not necessarily present in the DCIS samples during our analysis. It has been suggested that DCIS/T1 mic represents the earliest stage of neoplastic invasion and has a different biology than pure DCIS, although the molecular characteristics have not been identified [36].

We demonstrated in this study that in tissue measurements of Vav2 protein expression had discriminating power allowing for discernment of DCIS from DCIS with concurrent invasion. Hence, our novel findings suggest that Vav2 may be a companion diagnostic tool capable of predicting the likelihood of microinvasion that otherwise can be over- and under- diagnosed because of limitations with tissue sampling. Furthermore, Vav2 has been deemed a promising target for cancer therapy, with small molecule compounds being developed that specifically target Vav2 activity in cancer [37].

## Conclusions

This study describes the use of a novel imaging method for archival tissue testing, which may inform the status of protein BM in tumor and may help to stratify a women’s individual risk for tumor invasiveness to avoid potential over- or under-treatment. Despite the apparent limitation of our study DCIS cohort size, our novel findings on Vav2 hold promise for utilizing Vav2 protein as a BM of progressive DCIS and a target for cancer therapy.

## Electronic supplementary material

Additional file 1: Figure S1: Vav2 staining in positive (skin, placenta) and negative (lymph node) human tissue controls. Fresh 5 μm sections were cut from FFPE tissue blocks, de-paraffinized in xylene, rinsed in ethanol, and re-hydrated. Antigen was heat-retrieved in 10 mM Na citrate, pH6.0; endogenous peroxidase was quenched by pretreatment with 1.0% H_2_O_2_. Incubating with 5% goat serum minimized nonspecific staining. To detect specific protein, slides were incubated overnight at 4°C with commercially tested rabbit polyclonal Ab recognizing human Vav2 (H-200) purchased from Santa Cruz Biotechnology. A biotinylated goat anti-rabbit secondary Ab was used for detection bound primary Ab and as an isotype-matched negative control. Staining was developed for 4 min using the VECTASTAIN Elite ABC and the VECTOR VIP substrate kits (Vector Labs). Time of the development was optimized to avoid VIP saturation. Original magnification (x200). Arrows, examples of positively and negatively stained cells. Similar to the findings reported by the Swedish Human Protein Atlas Program (http://www.proteinatlas.org), the cells in lymph nodes were completely negative for Vav2 protein, whereas cells in the skin basal epidermal layer (*stratum basale*) and migratory trophoblasts in the placenta were intensely stained. (PDF 108 KB)
