# Supplementary figures and images for: Vav2 protein overexpression marks and may predict the aggressive subtype of ductal carcinoma in situ
Source: Biomark Res. 2014 Nov 28;2:22. doi: 10.1186/2050-7771-2-22 (PMC4362647; doi:10.1186/2050-7771-2-22)

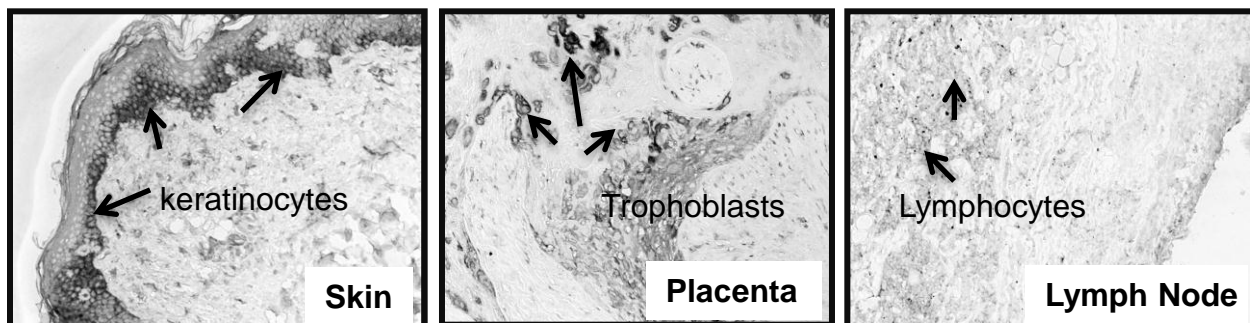

Vav2 IHC

Supplement: Supplementary file 1 — Additional file 1: Figure S1: Vav2 staining in positive (skin, placenta) and negative (lymph node) human tissue controls. Fresh 5 μm sections were cut from FFPE tissue blocks, de-paraffinized in xylene, rinsed in ethanol, and re-hydrated. Antigen was heat-retrieved in 10 mM Na citrate, pH6.0; endogenous peroxidase was quenched by pretreatment with 1.0% H2O2. Incubating with 5% goat serum minimized nonspecific staining. To detect specific protein, slides were incubated overnight at 4°C with commercially tested rabbit polyclonal Ab recognizing human Vav2 (H-200) purchased from Santa Cruz Biotechnology. A biotinylated goat anti-rabbit secondary Ab was used for detection bound primary Ab and as an isotype-matched negative control. Staining was developed for 4 min using the VECTASTAIN Elite ABC and the VECTOR VIP substrate kits (Vector Labs). Time of the development was optimized to avoid VIP saturation. Original magnification (x200). Arrows, examples of positively and negatively stained cells. Similar to the findings reported by the Swedish Human Protein Atlas Program (http://www.proteinatlas.org), the cells in lymph nodes were completely negative for Vav2 protein, whereas cells in the skin basal epidermal layer (stratum basale) and migratory trophoblasts in the placenta were intensely stained. (PDF 108 KB) [file 40364_2014_52_MOESM1_ESM.pdf]
